# How physicians identify with predetermined personalities and links to perceived performance and wellness outcomes: a cross-sectional study

**DOI:** 10.1186/s12913-014-0616-z

**Published:** 2014-11-29

**Authors:** Jane B Lemaire, Jean E Wallace

**Affiliations:** Faculty of Medicine, University of Calgary Health Sciences Center, 3330 Hospital Drive NW, Calgary, Alberta T2N 4N1 Canada; Department of Sociology, Faculty of Arts, University of Calgary, 2500 University Drive NW, Calgary, Alberta T2N 1N4 Canada

**Keywords:** Personality, Self-assessment, Health

## Abstract

**Background:**

Certain personalities are ascribed to physicians. This research aims to measure the extent to which physicians identify with three predetermined personalities (workaholic, Type A and control freak) and to explore links to perceptions of professional performance, and wellness outcomes.

**Methods:**

This is a cross-sectional study using a mail-out questionnaire sent to all practicing physicians (2957 eligible, 1178 responses, 40% response rate) in a geographical health region within a western Canadian province. Survey items were used to assess the extent to which participants felt they are somewhat of a workaholic, Type A and/or control freak, and if they believed that having these personalities makes one a better doctor. Participants’ wellness outcomes were also measured. Zero-order correlations were used to determine the relationships between physicians identifying with a personality and feeling it makes one a better doctor. T-tests were used to compare measures of physician wellness for those who identified with the personality versus those who did not.

**Results:**

53% of participants identified with the workaholic personality, 62% with the Type A, and 36% with the control freak. Identifying with any one of the personalities was correlated with feeling it makes one a better physician. There were statistically significant differences in several wellness outcomes comparing participants who identified with the personalities versus those who did not. These included higher levels of emotional exhaustion (workaholic, Type A and control freak), higher levels of anxiety (Type A and control freak) and higher levels of depression, poorer mental health and lower levels of job satisfaction (control freak). Participants who identified with the workaholic personality versus those who did not reported higher levels of job satisfaction, rewarding patient experiences and career commitment.

**Conclusions:**

Most participants identified with at least one of the three personalities. The beliefs of some participants that these personalities enhance professional performance may reinforce the harmful behaviors associated with poor wellness outcomes. Future research should further explore links between physician personality, perceptions of performance and actual performance, and more definitively address whether the perceived benefits offered by identifying with the workaholic personality are enough to counter the potential costs to physician wellness.

## Background

Personality has been defined as a collection of stable individual traits [1] and behavioral, emotional and attitudinal response patterns. Within the research literature, personality is described using various approaches. For example, personality dimensions or traits include designations of extraversion, introversion, neuroticism, agreeableness, conscientiousness, perfectionism, obsessionality, and low self-esteem [2,3]. Combinations of traits or personality types include categorizations of sensing, thinking, and judging types or impulsiveness and insecurity [4,5]. Personality components include labeled behaviors such as harm avoidance, novelty seeking, and reward dependence [6].

Personality research has been applied to the medical profession through the exploration of how certain features of physicians’ personalities relate to outcomes such as their career choice and patient care. For example, studies have documented trends in physicians’ personality types within particular medical specialties such as anesthesiology and emergency medicine [7,8]. Emotional stability and extraversion in physicians have been shown to predict job satisfaction [9]. In terms of patient care, certain personality traits affect the physician-patient relationship where, for example, physician openness and conscientiousness are linked to improved patient satisfaction [10] and patient-centered communication [11].

More generally, certain features of personality are often ascribed to physicians with, at times, conflicting inferences that relate to professional performance. For example, a driven, goal-oriented student is more likely to attain success in a medical career as these criteria are often used to judge admissibility to the profession. At the same time, the Type A personality frequently assigned to physicians holds many of the same attributes, but can be labeled undesirably as competitive, aggressive, or overachieving [12]. Similarly, physicians are rewarded throughout their careers for the pursuit of excellence, yet their determination to attain the unreachable “perfection” may promote behaviors that are then admonished as work addiction, the need to control all aspects of their work, or obsessive compulsivity [13]. The very nature of health care and medical professionalism may also reinforce this dissonance. Behaviors like conscientiousness, accessibility to patients, and the pursuit of excellence and lifelong learning are examples of the foundation tenets of medical professionalism [14]. Maintaining these professional standards while setting boundaries to preserve wellness is challenging for all physicians. The extreme application and/or distorted interpretation of these professional expectations can threaten physician wellness, and physicians with certain personalities may be at risk.

The “physician personality” is considered one of the individual characteristics that contributes, along with professional and organizational factors, to physician wellness [15]. Prior research has demonstrated how physicians’ personality traits are associated with their emotional wellness, where traits of neuroticism and conscientiousness are independent predictors of medical school stress for Norwegian medical students [16]. Personality has also been shown to facilitate or constrain effective coping responses [17] where extraversion and conscientiousness predicted greater use of problem-solving as a coping strategy. Certain personality traits such as compulsiveness put physicians at risk for burnout because of how they impact the individual’s response to workplace stressors [18] and internal medicine residents’ self-reported disorganized personality style at the beginning of training has been shown to be associated with the prevalence of burnout after one year [19]. The physician personality may also threaten the doctor’s physical health, where negative traits such as hostility have been linked to decreased survival, increased risk of developing cancer and coronary disease incidence [20-22].

Despite this body of literature, not much is known about whether or not physicians identify with certain personalities, if they perceive any link between these personalities and professional performance, and how they experience wellness when identifying with these personalities. During a qualitative study exploring the determinants of physician well-being, we noted that interviewed physicians frequently referred to themselves and to colleagues using three personality descriptions: workaholic, Type A and control freak [23]. The following are illustrative sample quotes:

“At present, my major source of stress is related to sorting out details related to integrating a research component into my career. The specific generators of stress relate to funding needs, high expectations and work being judged, and the nebulous nature of research work that potentially has no limit, requiring a Type A person to set the limits, and surrounded by other Type A people in the same boat.”

“I believe my stresses have decreased with the duration of my career due to: 1) wisdom: learning not to sweat the small stuff and delegate work 2) financial stability 3) confidence: I don’t need to be the workaholic that society and I expected me to be when I started my career…”

“I think it is necessary to be partly obsessive-compulsive, perfectionistic and a control freak when dealing with something as important as people’s lives/health.”

“I think physicians as a group just like, you know, are all Type A personalities and kind of go, go, go…”

In the literature, the workaholic personality has been described as one demonstrating a compulsion or uncontrollable need to work incessantly [24]; the Type A as one exhibiting competitiveness, perfectionism, anxiety, and high goal-orientation [25], and the control freak as one displaying attempts to dictate how everything around them is done [26]. To varying degrees, these personalities are also felt to be interlinked. For example, the anxious Type A personality emerges as a maladaptive coping response to deal with a high work stress environment; the control freak often exhibits perfectionism traits and Type A behaviors; and the workaholic frequently suffers from a form of obsessive compulsion or even addiction that helps mask anxiety [24-26]. These three personalities have also been linked to negative wellness outcomes. Workaholism has been identified as a risk factor for physician suicide [27] and perfectionism, a component of the Type A and control freak personalities, has been shown to be a vulnerability factor for depression, anxiety, burnout, and suicide [28-30].

The goal of this paper is to examine the extent to which physicians in our study identified with the workaholic, Type A and control freak personalities. Given that physicians’ personalities have been associated, at times with dissonance, with aspects of patient care and physician wellness, we compare those physicians who identify with these personalities versus those who do not in terms of two outcomes: how they perceive the impact of personality on professional performance and how they experience wellness as measured by emotional exhaustion, depression, anxiety, mental health, job satisfaction, rewarding patient care, and career commitment.

## Methods

### Setting and participants

Our cross-sectional study was conducted within what was, at the time of data collection, a single large health region in Western Canada that administered 43 health care centers and 12 acute care sites (including a major university teaching hospital, 3 additional urban hospitals, and 5 rural hospitals) and employed almost 3000 physicians. The questionnaires were mailed out to all physicians in the health region in March of 2008. We received 1178 surveys from an eligible 2957 physicians (40% response rate). Complete data on personality were available for 1151 respondents. The study was performed in collaboration with the health region, who administered the distribution and collection of the survey questionnaires. De-identified survey response data were provided to the study researchers along with demographic data including gender, age, and medical specialty. Information on non-responders was not available.

### Data sources/measurement

The survey items analyzed for this paper were part of a larger questionnaire administered in a mixed-methods study exploring the determinants of physician well-being [31]. The 8-page survey questionnaire asked physicians about their well-being, types of patients, work experiences, coping strategies, available supports, individual traits (the topic of this paper), and wellness. We measured physicians’ identification with predetermined personalities and their perceptions of the link to professional performance using three, two-part survey items (developed ad hoc by the authors) asking the extent to which participants agree or disagree (5-point Likert Scale) with the following: *It is accurate to say I am somewhat of a workaholic*, followed by: *having this trait makes one a better doctor*. The same items were asked substituting “Type A” and “control freak”. “Agree” and “strongly agree” responses were coded as describing oneself as having this personality and/or as believing this makes one a better doctor. “Disagree” and “strongly disagree” responses were coded as describing oneself as not having this personality and/or not believing this makes one a better doctor. “Neutral” responses were excluded from the analyses as our goal was to target those physicians that clearly did or did not identify with any of the three personalities and who expressed an opinion on a potential link between personality and professional performance assessment. We measured wellness outcomes using several items. Emotional exhaustion was measured using 5 items from Barnett et al’s revised version of the Maslach Burnout Inventory [32]: *I feel emotionally drained from my work; I feel used up at the end of the workday; I feel tired when I get up and have to face another day on the job; I feel that working all day is really a strain for me;* and *I feel burned out from my work* (response categories: never, not very often, sometimes, often, most of the time; coded 1–5 respectively; scores of all 5 items summed and divided by 5 to compute a mean score). Depression and anxiety were each measured using a single item from Williams et al. [33]: *I felt sad or depressed much of the time in the past year* and *I felt anxious or nervous much of the time in the past year* (response categories: never, almost never, sometimes, fairly often, very often; coded 1–5 respectively). Mental health was measured using a single item from Wallace [34]: *Compared with other people your age, how would you describe your mental or emotional health?* (response categories: poor, fair, good, very good, excellent; coded 1–5 respectively). Job satisfaction was measured using 3 items from Brayfield and Rothe [35]: *Most days I am enthusiastic about my job; I definitely dislike my job; I find real enjoyment in my job* (response categories: strongly disagree, disagree, neither agree nor disagree, agree, strongly agree; coded 1–5 respectively; scores of all 3 items summed and divided by 3 to compute a mean score). Rewarding patient interactions was measured using a single item from Wallace and Tighe [36]: *I find working with my patients very rewarding*, and career commitment was measured using a single item from Konrad et al. [37]: *If I were to choose over again, I would still become a physician* (both items’ response categories: strongly disagree, disagree, neither agree nor disagree, agree, strongly agree; coded 1–5 respectively).

### Statistical methods

We calculated the percentage of physicians who identified with each of the three personalities and what percentages of respondents viewed the personalities as relating positively or negatively to physician performance. To determine the relationship between physicians identifying with a personality and feeling it makes one a better doctor, zero-order correlations were calculated using Pearson’s correlation coefficient. T-tests were used to compare measures of physician wellness (burnout, depression, anxiety, good mental health, job satisfaction, rewarding patient care, and career commitment) for physicians who identified with the personality vs. those who did not. Sensitivity analyses were conducted using non-parametric tests (Wilcoxon rank-sum and Spearman’s correlation coefficient). Sensitivity analyses were also conducted using different definitions of identifying with a certain personality type (“strongly agree” only, versus “disagree/strongly disagree”) and by grouping the “neutral” responses alternately with the “agree/strongly agree” or “disagree/strongly disagree” category. Statistical significance was defined as p < 0.05, and p-values were two-sided.

Ethics approval for this study was obtained from the Conjoint Health Ethics Review Board of the University of Calgary.

## Results

### Participants

Of the physicians who completed the questionnaire items relevant to this analysis, 653 (58%) were men and 474 (42%) were women. Our respondents were, on average, 49 years of age (range = 27-89 years) and practiced medicine for about 18 years. Most of the participants participated in on-call rotations (77%) and half (50%) were involved in a group practice. They reported working, on average, approximately 50 hours per week when engaged in mainly patient-care related duties (excluding on-call hours), although this varied greatly. The participant sample is proportionally representative of the physicians in the health region in terms of their medical specialty (Table 1).

**Table 1:** **Breakdown of physicians’ medical specialty for health region (June 2008) versus survey sample (March 2008)**

***Medical specialty***	***Health region N (%)***	***Survey sample N (%)***
Anesthesiology	145 (6%)	73 (6%)
Clinical Neurology	72 (3%)	18 (2%)
Diagnostic Imaging	83 (4%)	22 (2%)
Emergency	87 (3%)	58 (5%)
Family Medicine	772 (31%)	407 (33%)
Internal Medicine	447 (18%)	199 (17%)
Obstetrics/Gynecology	68 (3%)	24 (2%)
Pathology	66 (3%)	26 (2%)
Pediatrics	198 (8%)	78 (7%)
Psychiatry	123 (5%)	73 (6%)
Rural Medicine	181 (7%)	82 (7%)
Surgery	220 (9%)	117 (10%)
Other		83 (7%)
**TOTAL**	**2488 (100%)**	**1178 (100%)**

### Personality and perceived professional performance

**Figure 1:**
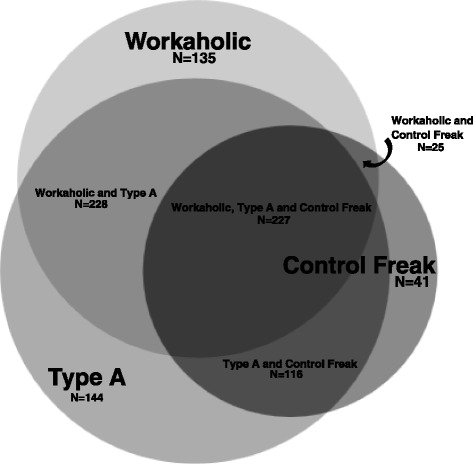
**Proportions of 916 respondents who identified with one, two, or all three of the predetermined personalities.**

Table 2 and Figure 1 summarize how many physicians identified with one and/or a combination of the three personalities. About half (53%) of the participants saw themselves as workaholics, two thirds (62%) as Type As and one third (36%) as control freaks. Many identified with a combination of personalities such as workaholic and Type A (19%), Type A and control freak (10%), or workaholic and control freak (2%). Some (19%) identified with all three, and some (22%) with none. Table 2 also shows how participants linked these personalities to professional performance. While half (52%) the participants felt that being a Type A improves performance, only 37% and 18% respectively felt that being a workaholic or control freak did so. A sub-group analysis was performed and determined that participants who identified with a particular personality versus those who did not were significantly more likely to think it improves professional performance for all three personalities and vice-versa (Table 2). Specifically, half (55%) of the participants who identified with the workaholic personality (compared to 13% who did not), three quarters (72%) of those identifying with the Type A personality (compared to 9% who did not), and 40% of those identifying with the control freak personality (compared to 5% who did not) felt that the personality improves professional performance. The correlations between identifying with a personality and the perception that it makes one a better physician were moderate across the three personalities (workaholic Pearson’s r = .504, p < 0.001; Type A Pearson’s r = .605, p = < 0.001 and control freak Pearson’s r = .571; p < 0.001) Sensitivity analyses conducted using Spearman’s r for non-parametric data yielded similar results.

**Table 2:** **Percentage of physicians who identify with personalities and links to perceived performance**

**How physicians describe themselves**							
	**Responses**	Report this personality*	**Do not report this personality†**	**Report a combination of personalities N (%)**			
	**N**	**N (%)**	**N (%)**				
Workaholic	1150	615 (53)	318 (28)	Workaholic and Type A	228 (19)	All three traits	227 (19)
Type A	1148	715 (62)	204 (18)	Type A and control freak	116 (10)	None of the three traits	238 (21)
Control freak	1149	409 (36)	474 (41)	Workaholic and control freak	25 (2)		
**How physicians view the personalities in terms of professional performance**							
	**Responses**	**Feel it makes one a better doctor‡**	**Do not feel it makes one a better doctor§**	**Report this personality and feel it makes one a better doctor**	**Do not report this personality but feel it makes one a better doctor**	**Correlations between reporting the personality (or not) and feeling it makes one a better doctor (or not)**	
	**N**	**N (%)**	**N (%)**	**N (%)**	**N (%)**	**(p value)**	
Workaholic	1132	418 (37)	433 (38)	328/602 (55)	40/307 (13)	.504 (<0.001)	
Type A	1130	586 (52)	258 (23)	507/705 (72)	17/194 (9)	.605 (<0.001)	
Control freak	1131	205 (18)	651 (58)	162/402 (40)	21/455 (5)	.571 (<0.001)	

*Strongly agree or agree with the statement: “It is accurate to say I am somewhat of a…”

†Strongly disagree or disagree with the statement: “It is accurate to say I am somewhat of a…”

‡Strongly agree or agree with the statement: “ Having this trait makes one a better doctor”

§Strongly disagree or disagree with the statement: “ Having this trait makes one a better doctor”

### Personality and wellness outcomes

Table 3 compares the mean scores of the seven wellness outcomes for physicians who identified with the workaholic, Type A and control freak personalities versus those who did not. Those who identified with the workaholic personality reported significantly higher levels of emotional exhaustion (2.93 vs. 2.75; p = .001), but also higher levels of positive outcomes including job satisfaction (4.07 vs. 3.97; p = .032), rewarding patient experiences (4.23 vs. 4.10; p = .001) and career commitment (4.00 vs. 3.81; p = .006) than those who did not. Those who identified with the Type A personality reported significantly higher levels of emotional exhaustion (2.92 vs. 2.73; p = .002) and anxiety (2.91 vs. 2.58; p = .000) than those who did not. Those who identified with the control freak personality reported significantly higher levels of emotional exhaustion (3.03 vs. 2.69; p = .000), depression (2.71 vs. 2.47; p = .000), and anxiety (3.01 vs. 2.60; p = .000), and lower levels of good mental health (3.41 vs. 3.65; p = .000) and job satisfaction (3.96 vs. 4.07; p = .011) than those who did not. Sensitivity analyses conducted using the Wilcoxon rank-sum test for non-parametric data yielded similar results, as did analyses using only “strongly agree” to denote identification with a personality, as well as analyses grouping the “neutral” responses alternately with the “agree/strongly agree” or “disagree/strongly disagree” category (results available on request).

**Table 3:** **Comparison of wellness outcomes for physicians who identified with the workaholic, Type A and control freak personalities versus those who did not**

***Higher scores indicate higher levels of outcomeMean scores (SD)***	**Workaholic N = 615**	**Not a workaholic N = 318**	**p value**	**Type A N = 715**	**Not a Type A N = 204**	**p value**	**Control freak N = 409**	**Not a control freak N = 474**	**p value**
**Emotional exhaustion**	2.93 (.802)	2.75 (.756)	.001	2.92 (.802)	2.73 (.773)	.002	3.03 (.814)	2.69 (.748)	.000
**Depression**	2.64 (.865)	2.57 (.845)	.234	2.64 (.864)	2.51 (.888)	.053	2.71 (.893)	2.47 (.861)	.000
**Anxiety**	2.84 (.883)	2.77 (.834)	.243	2.91 (.883)	2.58 (.860)	.000	3.01 (.875)	2.60 (.853)	.000
**Good mental health**	3.52 (.945)	3.54 (.835)	.823	3.51 (.907)	3.55 (.900)	.521	3.41 (.942)	3.65 (.874)	.000
**Job satisfaction**	4.07 (.637)	3.97 (.620)	.032	4.04 (.648)	4.00 (.607)	.510	3.96 (.658)	4.07 (.625)	.011
**Rewarding patient experiences**	4.23 (.582)	4.10 (.580)	.001	4.16 (.567)	4.18 (.649)	.705	4.14 (.555)	4.19 (.627)	.270
**Career commitment**	4.00 (.991)	3.81 (.995)	.006	3.94 (1.067)	3.86 (1.049)	.807	3.87 (1.030)	3.96 (1.026)	.208

## Discussion

Most of the physicians in our study identified with at least one of the workaholic, Type A, and/or control freak personalities. The Type A personality was most frequently linked to being a better doctor, although participants who identified with any of the three personalities were more likely to believe that the particular personality enhanced professional performance. Wellness outcomes differed for physicians who identified with a particular personality compared to those who did not. Most strikingly, identifying with the workaholic personality was associated with only one potentially harmful and three positive wellness outcomes, while identifying with the control freak personality was associated with five potentially harmful and no positive wellness outcomes.

We anticipated that these three personalities would resonate with our survey participants to some degree given our previous qualitative work where physicians described themselves and their colleagues in this way [23]. Our results may also reflect that individuals who choose a medical career possess personality traits associated with workaholic, Type A, and control freak personalities (e.g. ambitious, hard-working, obsessive-compulsive, perfectionistic). Prior research supports this notion in which, for example, low self-esteem was shown as a personality antecedent of burnout in physicians [38], and internal medicine residents, compared with the general population, were more likely to have higher aspiration levels [39]. Medical school admission criteria may also select directly or indirectly for certain personalities. One study demonstrated a positive association between extraversion and potential candidates’ multiple mini-interview performance and between extraversion/agreeableness and their acceptance offers [40]. It is possible that other personality traits associated with the workaholic, Type A and control freak personalities are also linked to medical school admission (e.g., high-goal orientation, competitiveness). A further potential explanation of our study results is that of physicians’ indoctrination into the culture of medicine and stressful work environment as shaping their coping strategies towards the behaviors exhibited by those with workaholic, Type A [12], and/or control freak personalities, or exposing and reinforcing the maladaptive aspects of the three personalities.

The physicians in our study linked physician personality to performance where the majority felt that being Type A makes one a better doctor and identifying with any of the three personalities was correlated with feeling it enhanced professional performance. To our knowledge, there is very little literature that explores this link. In a previous study of physician wellness, we explored physicians’ views of how their colleagues link their personal wellness to their ability to deliver quality health care. The interviewed physicians felt that despite an intellectual acknowledgment that wellness is associated with professional performance, two potential factors deterred physicians from recognizing signs of being unwell and caring for themselves: 1) the culture of medicine in which physicians are seen as invincible caregivers, highly committed to their patients, careers and sense of professionalism, and 2) physicians’ workloads, with external workplace pressures and the overwhelming nature of their work [41]. These two powerful professional influences may create a similar dissonance for physicians thinking about how personality relates to performance. They may view aspects of the workaholic, Type A, and control freak personalities through the sometimes distorting lenses of the culture of medicine and their incessant workload. As examples, workaholism has been described as a “respectable addiction”, and an expression of career devotion [42]. The Type A physician who is organized, proactive, and time efficient may be forgiven for his or her irritation and hostility by colleagues who appreciate his or her work effectiveness. The physician with a control freak personality may justify their extreme obsessiveness and need to have things done right as upholding professional cultural norms. Further research is needed to explore physicians’ perceptions of the link between personality and professional performance.

In addition, we found that wellness outcomes differed for participants who identified with the workaholic, Type A or control freak personalities versus those who did not. Prior research has shown that these personalities are associated with emotional exhaustion [38,43,44]. There is also a proven association between burnout and long work hours [15,45] - an occupational reality potentially aggravated by behaviors associated with the three personalities. In our study, identifying with the workaholic personality was associated with several positive wellness outcomes and further research could test these associations in a study designed to assess causality. It would also be important to explore if greater job satisfaction, rewarding patient experiences, and career commitment are drivers or consequences of identifying with the workaholic personality, and whether these perceived benefits are enough to counter the potential costs. Identifying with the control freak personality was associated with only negative wellness outcomes. Recall that physicians were also least likely to associate the control freak personality with improved performance. Future research might delve further into these insights.

Our results should be interpreted in light of the study design. This is a cross-sectional study in which physicians reported the extent to which they identified with certain personalities, rather than undergoing a formal personality assessment. Prior research supports that study participants are able to predict outcomes of personality testing [46], thus the responses of the physicians in our study may have some degree of validity and at the very least reflect their self-perceptions. We also limited this study to three personalities, thus it is not possible for us to know how other personalities would be relevant to these particular outcomes. It is also feasible that the interpretation of the terms workaholic, Type A, and control freak might mean different things to different individuals. However, this choice was deliberately informed by our previous qualitative data where interviewed physicians described themselves and their colleagues using this wording. There may also be a response bias in that physicians who completed our survey may have been more or less interested in physician wellness which was the focus of the study. Additionally, the fact that there are no data to allow consideration of nonresponse bias is an important limitation, particularly in light of the 40% response rate. Also, only the emotional exhaustion domain of burnout was assessed as it appeared to be the best understood and critical in understanding the burnout process at the time. Future research could explore how the depersonalization domain of burnout might link to these personalities [47]. Although this bivariate analysis has revealed some interesting findings, these concepts need to be explored more empirically. Such studies could expose how confounding factors such as race, career stage, gender and specialty may affect the associations between personality type and the two key outcomes discussed in this paper. Also important to note is that despite statistical significance, the absolute differences in wellness outcomes between those respondents who identified with a personality compared with those who did not were generally small and do not meet previously proposed criteria of half a standard deviation to denote clinical significance [48]. Although hypothesis generating, these findings deserve further study to assess clinical relevance.

## Conclusions

The existing literature supports that physicians working in the demanding medical profession are at substantial risk of being unwell with negative personal and professional consequences [15,45]. The physicians in our study who identified with the workaholic, Type A, and control freak personalities, more so than those who did not, believe that these personalities enhance professional performance. These beliefs, along with the sometimes dissonant messaging from the culture of medicine and the physician workplace, may reinforce the harmful behaviors associated with these personalities. Our exploratory study also demonstrates small absolute differences in health outcomes between those two groups of physicians. Though hypothesis generating, further research would help clarify if these are clinically meaningful differences and further explore links between physician personality, perceptions of performance, and actual performance. Looking forward, a simple intervention asking physicians the extent to which they identify with the workaholic, Type A and control freak personalities may offer the opportunity to recognize those at increased risk, enhance their awareness of potential harm, and teach strategies that could lessen the damaging effects, while maintaining any potentially beneficial adaptive elements of these personality traits.
